# Nanotherapy for Cancer and Biological Activities of Green Synthesized AgNPs Using Aqueous *Saussurea costus* Leaves and Roots Extracts

**DOI:** 10.3390/ph17101371

**Published:** 2024-10-15

**Authors:** Mina A. Almayouf, Raihane Charguia, Manal A. Awad, Abir Ben Bacha, Imen Ben Abdelmalek

**Affiliations:** 1 Department of Biology, College of Science, Qassim University, Buraydah 51452, Saudi Arabia; mm.abdulmalek@qu.edu.sa; 2 Department of Physics, College of Science, Qassim University, Buraydah 51452, Saudi Arabia; 141296@qu.edu.sa; 3 King Abdullah Institute for Nanotechnology, King Saud University, Riyadh 11451, Saudi Arabia; mawad@ksu.edu.sa; 4 Department of Biochemistry, College of Science, King Saud University, P.O. Box 22452, Riyadh 11495, Saudi Arabia

**Keywords:** nanotechnology, green biotechnology, anticancer therapies, medicinal plants

## Abstract

**Background/Objectives**: Nanoparticles derived from medicinal plants are gaining attention for their diverse biological activities and potential therapeutic applications. **Methods**: This study explored the antioxidant, anti-inflammatory, anti-tumoral, and antimicrobial properties of green synthesized silver nanoparticles (AgNPs) using the aqueous leaf and root extracts of *Saussurea costus* (*S. costus*). The physicochemical characterizations of both biosynthesized AgNPs using the aqueous leaf extract (L-AgNPs) and root extract (R-AgNPs) were examined using UV spectroscopy, fluorescence spectroscopy, transmission electron microscopy, energy-dispersive X-ray spectroscopy, X-ray diffraction, dynamic light scattering, and Fourier-transform infrared spectroscopy. The antioxidant activity measured using ABTS, DPPH, and FRAP assays showed that AgNPs, particularly from roots, had higher activity than aqueous extracts, attributed to phenolic compounds acting as capping and antioxidant agents. **Results**: Enzyme inhibition studies indicated that AgNPs exhibited remarkable anti-inflammatory effects, inhibiting COX-1, 5-LOX, and secreted PLA_2_ enzymes by over 99% at 120 µg/mL, comparable to standard drugs. The anti-tumoral effects were evaluated on the human cancer cell lines HCT-116, LoVo, and MDA-MB-231, with AgNPs inhibiting cell proliferation dose-dependently and IC_50_ values between 42 and 60 µg/mL, demonstrating greater potency than extracts. The AgNPs also showed enhanced antimicrobial activities against various microbial strains, with IC_50_ values as low as 14 µg/mL, which could be linked to nanoparticle interactions with microbial cell membranes, causing structural damage and cell death. **Conclusions**: These findings suggest that *S. costus*-derived AgNPs are promising natural, biodegradable agents for various biological applications and potential new therapeutic agents, necessitating further research to explore their mechanisms and applications.

## 1. Introduction

The focus of nanotechnology is the creation of nanoparticles with regulated dispersity, size, shape, and chemical composition that may have applications in human health. Well-defined, pure nanoparticles can be produced with success using physical and chemical techniques, but these processes are costly and may pose environmental risks [1,2,3]. Various techniques have been devised to generate nanoparticles, with chemical approaches being the most widely used. Nevertheless, certain chemical methods need the use of harmful compounds in their synthesis protocol, which cannot be avoided. Ever since noble nanoparticles, including platinum, silver, and gold, were used for human contact on a mass scale, there has been a growing desire to develop environmentally benign synthesis procedures and avoid utilizing harmful chemicals when creating nanoparticles [4]. Green synthesis techniques offer an achievable choice by using bio-based materials such as plants, microorganisms, and agricultural waste as environmentally benign sources for nanoparticle production [5,6].

The diversity and richness of plant species offer a rich source of bioactive chemicals for the synthesis of nanoparticles, which has attracted significant attention to plant-mediated synthesis [7,8]. The capacity of plant components like leaves, roots, and seeds to lower metal ions and promote the creation of nanoparticles has been investigated [9]. Additionally, a number of factors, including ease of handling, obstruction of cell maintenance, the ability to quickly obtain a large number of plant extracts, and the fact that plant material is the best reduction agent for obtaining excellent nanoparticle size and shape, suggest that using plant extracts for nanoparticle synthesis could be advantageous over other sources [10].

Because of their biocompatibility and regulated release of chemicals, green-synthesized nanoparticles have a wide range of applications [11,12]. These nanoparticles have a lot of potential for use in drug delivery systems [13,14]; cancer treatment [15,16]; antimicrobial, antiparasitic, anti-inflammatory, and antioxidant effects; and other biomedical fields [9,17]. A safe way to conserve energy, stay away from organic solvents, and prevent the possibility of producing nanoparticles on a wide scale is to use green synthesis to create silver nanoparticles, or AgNPs [18,19]. Specifically, plant material (whole plants, leaves, stems, or roots) is used in plant-mediated production of AgNPs to reduce and stabilize silver ions into silver nanoparticles [20]. In addition to being easily accessible, inexpensive, low maintenance, and environmentally benign, plants can be handled securely [21].

AgNPs are described as nanomaterials having all their dimensions falling between 1 and 100 nm. When compared with bulk silver, these have demonstrated a higher surface area-to-volume ratio and larger capacity. Due to the material’s distinctive electrical, optical, and catalytic capabilities at the nanoscale, devices for targeted medication delivery, diagnosis, detection, and imaging have been researched and manufactured [22]. Of the several metallic nanoparticles that have been generated thus far, silver nanoparticles (AgNPs) have garnered significant interest within the biomedical industry. Equally, the improving effect of AgNPs on the fluorescence observation of pharmaceuticals has been the subject of numerous published studies [23].

The *Saussurea costus* (Falc.) (*S. costus*) Clarke family Asteraceae is a large, widely dispersed family with around 1000 generations [10,24]. Various species are grown in the Himalayas [25], Pakistan [26], and India [27]. In Arab nations, *Saussurea costus* is called “Al Kost Al-Hindi” and is utilized in Islamic medicine [28]. *S. costus* is a well-known medicinal plant that has been widely used in many ethnic medicines to treat a wide range of conditions, including microbial infections, thyroid disorders, ulcers, stomach issues, inflammatory diseases, and asthma [29,30,31]. The *S*. *costus* root extract contains a variety of bioactive phytoconstituents, with polyphenols, sesquiterpene lactones, alkaloids, essential oils, triterpenes, lignans, and tannins being the primary ones [32,33]. It has been declared that the active ingredients in *S. costus* root have effective anti-inflammatory, anticancer, antioxidant, antiulcer and hypoglycemic activity capabilities. Silver [34], magnesium [26], selenium [30], and zinc [12] oxides are synthesized using the plant extract *S. costus*. Synthesized nanoparticles are also utilized for various biotechnological applications, including environmental degradation of dye contaminants [10] and antimicrobial and antifungal cytotoxicity [26,30,35].

The current study investigated using the *S. costus* leaf and root aqueous extract as a reducing and capping agent for the green synthesis of AgNPs. A high-yielding synthesis procedure that produced uniform, monodispersed distributions, long-term stability, and spherical-shaped AgNPs was efficiently achieved by optimizing the synthetic parameters, which included temperature, reaction duration, and reagents’ concentrations. The nanoparticles’ catalytic activities were assessed by evaluating their ability to inhibit some key inflammatory enzymes, including COX, 5-LOX, and sPLA_2_s. The in vitro antioxidant, antimicrobial, and anticancer potential of the biologically synthesized AgNPs is also reported in this study.

## 2. Results and Discussion

### 2.1. Microstructural and Optical Studies

**Figure 1:**
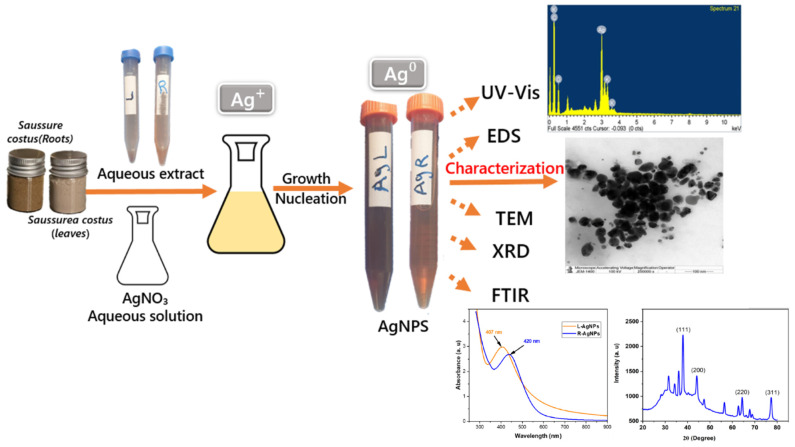
The possible formation mechanism of Ag nanoparticles using *S. costus* leaf and root extracts.

Figure 1 depicts the reaction wherein plant extracts from both leaves and roots of *S. costus* were individually mixed with a 1 mM silver nitrate solution, resulting in a change in color from yellow to dark brown, indicative of nanoparticle formation. Silver nanoparticles typically exhibit a yellowish-brown hue when suspended in water. Structurally, significant color alterations were observed in the *S. costus* extracts, further confirmed through UV spectrophotometry, as illustrated in Figure 1. The shift in color from yellow to dark brown in the reaction mixture provides a tangible indication of nanoparticle formation, attributable to the surface plasmon resonance (SPR) of the synthesized AgNPs. Similar results were observed with extracts from the Myrtaceae family, *Polyalthia longifolia* leaves, *Hypsizygus ulmarius*, *Leea macrophylla* leaf extract, and *Annona squamosa* fruit and leaves [36,37,38,39].

**Figure 2:**
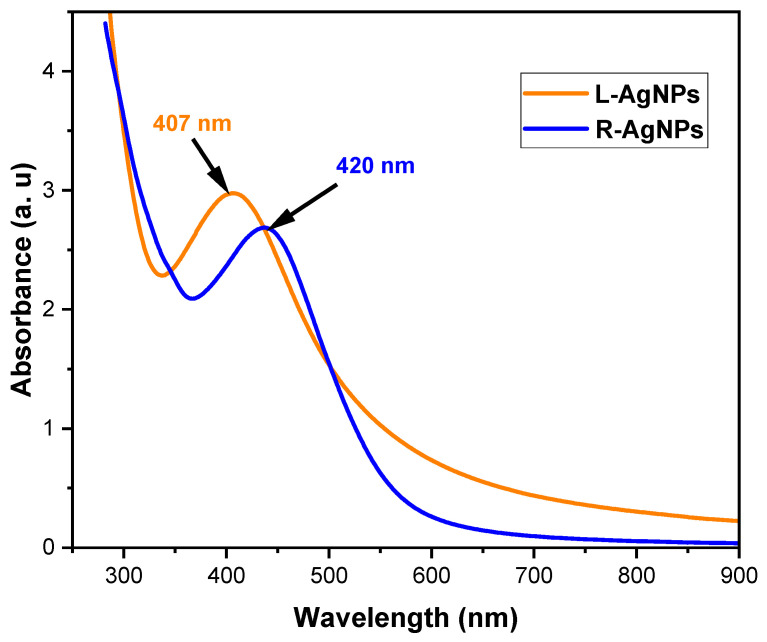
UV analysis spectra of L-AgNPs and R-AgNPs.

The UV–Vis absorption spectra of the synthesized silver nanoparticles are presented in Figure 2. UV–Vis spectroscopy is a widely used method for nanoparticle analysis, where free electrons within the silver nanoparticles induce surface plasmon resonance (SPR). The synthesized AgNPs using *S. costus* leaves (L-AgNPs) exhibited a visible absorption peak (SPR) centered at 407 nm, while the synthesized AgNPs using *S. costus* roots (R-AgNPs) displayed one at 420 nm. This specific SPR, due to its collective oscillation resonating with light waves, is a characteristic band for Ag [40,41]. In the visible spectrum, this band corresponds to the absorption of AgNPs resulting from external plasmon sensing [42]. The strengthened peaks demonstrate enhanced absorption of AgNPs, with the symmetrical and narrow absorption peak indicating an ordered size distribution of the AgNPs. As the maximum absorption wavelength experiences pronounced shifts with time, it becomes evident that the size of the AgNPs decreases, considering the rapid increase in both the nuclei burst rate and nuclei generation during core rupture over extended periods. In essence, as the final particle count rises, the variance in particle dimensions diminishes. This analysis underscores the potential of *S. costus* leaf extracts to reduce AgNPs [43]. Silver nanoparticles often exhibit UV peaks in the 400–500 nm wavelength range, depending on their size, as documented in the literature. Further investigation entailed morphological TEM analysis, confirming the formation of approximately spherical-shaped AgNPs in response to a single absorption peak, indicative of surface plasmon resonance (SPR) induced by unbound electrons in metal nanoparticles vibrating in resonance with light waves [44,45,46].

**Figure 3:**
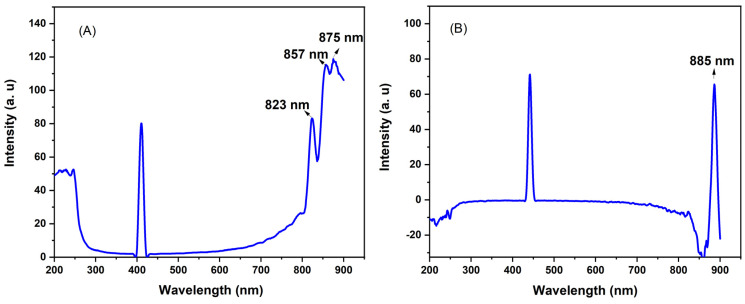
Fluorescence emission spectra of (**A**) L-AgNPs and (**B**) R-AgNPs.

We investigated the optical properties of the L-AgNPs and R-AgNPs in distilled water using fluorescence spectroscopy (Figure 3). The colloidal silver nanoparticles were dispersed in water and their emission spectra analyzed at excitation wavelengths of 407 nm and 420 nm for L-AgNPs and R-AgNPs (Figure 3A,B), respectively. Emission measurements across the spectral range from 200 to 900 nm revealed a slight rightward shift in the absorption peak toward higher wavelengths. The fluorescence emission peaks corresponded to relaxation processes involving surface plasmons and electron recombination [47]. In Figure 3B, the fluorescence emission peak observed at 885 nm suggests a uniform size distribution of the R-AgNPs. When examining the excitation wavelengths of the L-AgNPs (Figure 3A), it was noted that three peaks at 823, 857, and 875 nm were present. The presence of various particle sizes within the sample could influence these peaks. This observation suggests a blue shift in the emission peak as particle size decreases, which is linked to increased nanoparticle interaction [48]. The fluorescence process involves photoexcitation, electron relaxation, and emission. While silver nanoparticles hold promise for optical applications, understanding photoluminescence mechanisms remains a challenge [49,50].

Transmission electron microscopy (TEM) was employed to determine the size, shape, and morphology of the nanoparticles. This technique allows researchers to assess the distribution of nanoparticles, their structural properties, and the dominant particle sizes. The majority of the silver nanoparticles appeared uniform in shape and size, although a small proportion exhibited non-isomorphic structures [51]. 

**Figure 4:**
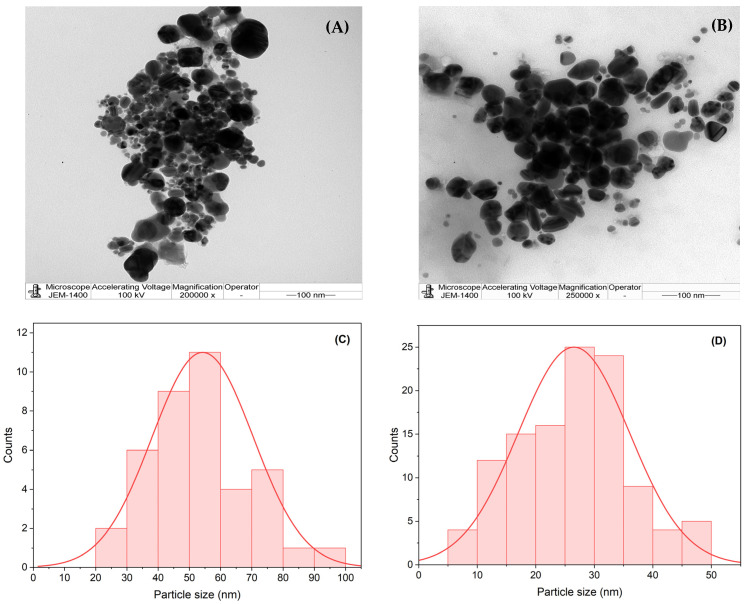
TEM images for the synthesized L-AgNPs (**A**) and R-AgNPs (**B**). Histograms of the particle size distributions of L-AgNPs (**C**) and R-AgNPs (**D**).

Figure 4A,B display TEM images of L-AgNPs and R-AgNPs, respectively, revealing a variety of nanoparticle morphologies, including spherical, semi-spherical, irregular, rod-like, and cubic forms. The nanoparticles were monodispersed, showing no significant agglomeration and indicating an even distribution of shapes. It was observed that biomolecules might have coated the R-AgNPs, as evidenced by their lighter edges compared with their centers. Additionally, the results indicated that different plant extracts, both in the presence and absence of element ions, produced varied nanoparticle morphologies [52]. The TEM images were assessed with Image J software (v.1.54g), as reported in the histograms (Figure 4C,D). An approximate particle size of 55 nm for the L-AgNPs and 26 nm for the R-AgNPs was determined by applying the Gaussian function for histogram fitting.

**Figure 5:**
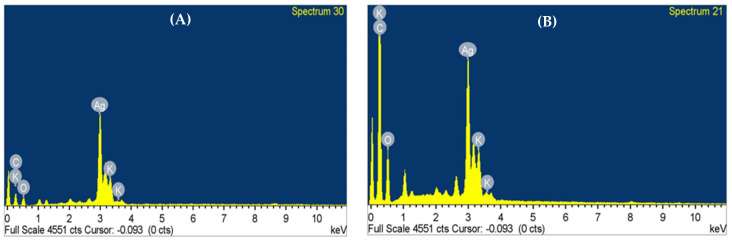
EDX spectra of (**A**) L-AgNPs and (**B**) R-AgNPs.

Nanoparticle purity and elemental composition were confirmed using energy-dispersive X-ray spectroscopy (EDS) and core mapping. Figure 5A,B illustrate that the generated nanoparticles possess an energy-dispersive spectrum, indicating the presence of silver. Metallic silver nanoparticles typically exhibit a strong signal peak around 3 keV due to surface plasmon resonance [53,54]. Quantitative EDX analysis of the AgNPs revealed strong signals of Ag atoms for both synthesized samples, as depicted in Figure 5. The reduction of silver ions using *S. costus* leaf and root extracts results in crystalline nanoparticles, as evidenced by the EDX pattern. Bioactive molecules attached to the surfaces of the silver nanoparticles may have contributed additional elements such as carbon (C), potassium (K), and oxygen (O) to the prepared samples of L-AgNPs and R-AgNPs (Figure 5). Nanoparticles derived from plant extracts have this advantage over their synthetically produced counterparts due to the incorporation of these additional elements. One possible explanation for the presence of these peaks may be the use of carbon tape and organic components during the measurement process. Previous research indicated that *Artemisia nilagirica* leaf extract and *Artocarpus heterophyllus* seed extract can generate peaks in the 2–4 keV range from silver nanoparticles [55].

**Figure 6:**
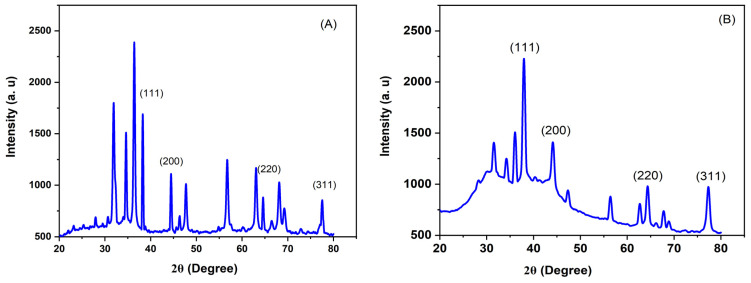
XRD spectra of (**A**) L-AgNPs and (**B**) R-AgNPs.

X-ray diffraction (XRD) is a reliable method to study the evolution of silver nanoparticles (AgNPs), determine the size of crystalline particles, and understand the synthesizing process. Figure 6A displays the XRD pattern of the L-AgNPs, showing four sharp peaks at 38.121°, 44.308°, 64.458°, and 77.415°, corresponding to the face-centered cubic (fcc) planes (111), (200), (220), and (311), respectively. Similarly, Figure 6B shows the XRD pattern of the R-AgNPs with peaks at 38.116°, 44.301°, 64.448°, and 77.4°, indicating a face-centered cubic structure with the $Fd\bar{3}m$ space group (N Ɗ 225), confirming the successful synthesis of R-AgNPs. These findings are consistent with previous data, indicating a promising trend [56]. The lattice constant α = 4.079 Å for L-AgNPs was derived from the pattern, confirming the reference fcc structure (COD 1100136). Consistent with results reported in previous studies, metabolites covering AgNPs may be responsible for the additional spectrum peaks observed for L-AgNPs [57,58].

To determine the size of AgNPs, the Debye–Scherrer equation $D=\frac{k\lambda }{\beta cos\theta }$ was used.

The factors being examined were the size of the AgNP crystallites (D); the wavelength of the X-ray source (λ) (1.54060 Å); the width of the diffraction peak at half of its highest intensity (FWHM), in radians; the Scherrer constant (k), which varies from 0.9 to 1; and the Bragg angle (θ), in radians.

Based on the XRD pattern analysis, the crystalline sizes of the L-AgNPs and R-AgNPs were calculated to be 19 nm and 11 nm, respectively, considering the X-ray wavelength of 1.5418 Å and the FWHM and Bragg diffraction angle of the dominant peak. For the R-AgNPs sample, the lattice constant was found to be 4.08550 Å, consistent with the standard value of a = 4.086 Å (JCPDS File No. COD 1100136).

**Figure 7:**
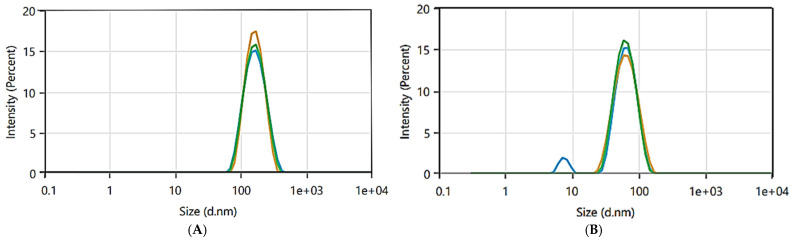
DLS analysis of (**A**) L-AgNPs and (**B**) R-AgNPs.

Dynamic light scattering (DLS) is a versatile method for nanoparticle identification and sizing. It measures fluctuations in scattered light from dispersed nanoparticles over time, which result from Brownian motion. The hydrodynamic diameters, which correspond to the nanoparticle size distribution, determine the frequency of these oscillations. The dimensionless polydispersity index (PDI) is used to estimate the homogeneity of particle size distribution and nanoparticle aggregation [59]. Figure 7A shows that the average size of the L-AgNPs was 148.8 nm, with a narrow particle size distribution. The data indicated an intercept of 0.9815 and a PDI of 0.1448. The zeta nanoparticle size analyzer yielded larger sizes than those obtained from TEM. PDI values range from 0 to 1; values of less than or equal to 0.3 suggest that the AgNPs are likely monodispersed and have a homogeneous distribution, while PDI values greater than 0.7 indicate a highly polydisperse particle size distribution, which is not ideal for DLS measurement [60]. In contrast, Figure 7B shows the zeta volume as a function of density (%) for the R-AgNPs. The average size of the composite nanoparticles was 50.32 nm for the R-AgNPs. The recorded PDI value was 0.2578 for the R-AgNPs, indicating monodisperse distributions and long-term stability. We observed that the particle size determined by DLS was greater than the dimensions seen via TEM. Yet the particle size identified through dynamic light scattering (DLS) is influenced not only by the metallic core of AgNPs but also by the stabilizing agents present on the nanoparticles’ surfaces [50,59].

**Figure 8:**
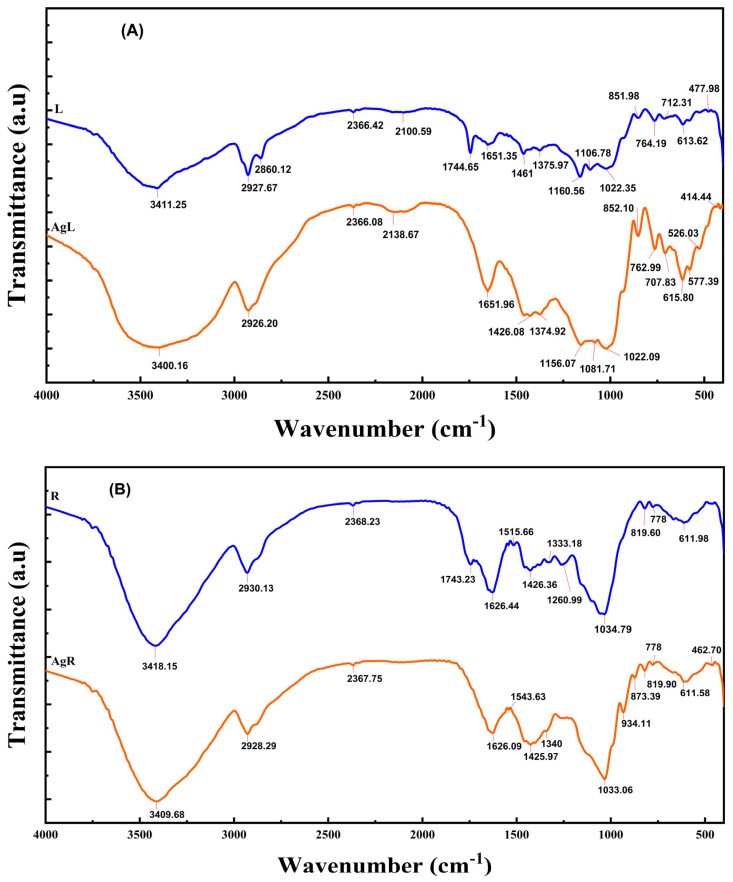
FTIR spectra of (**A**) L-AgNPs and *S. costus* leaf extract (**B**) R-AgNPs and *S. costus* root extract.

The purpose of performing FTIR analysis on the plant extract was to identify functional groups and ascertain their potential contribution to the synthesis and stability of the silver nanoparticles. By analyzing the prepared silver nanoparticles using Fourier transform infrared spectroscopy, we verified the presence of specific functional groups and the dual function of the plants as reducing and capping agents. Differences in peak intensity or wavenumber changes may indicate the functional groups involved in the cross-linking processes. These absorption bands were observed at 3411.25, 2927.67, 2860.12, 2366.42, 2100.59, 1744.65, 1651.35, 1461.00, 1375.97, 1160.56, 1106.78, 1022.35, 851.98, 764.19, 712.31, 613.62, and 477.98 cm^−1^ in the FTIR spectra of *S. costus* leaf extract. In Figure 8A, the absorption bands are at 3400.16, 2926.20, 2366.08, 2138.67, 1651.96, 1426.08, 1374.92, 1156.07, 1081.71, 1022.09, 852.10, 762.99, 707.83, 615.80, 577.39, 526.03, and 414.44 cm^−1^ in the FTIR spectra of the L-AgNPs.

There was a slight difference in the binding sites between the extracted AgNPs and the synthesized AgNPs in terms of IR spectra, indicating that the biomolecules were adsorbed on the silver. The stretching vibration peak ν(OH), which may indicate the presence of carbohydrates or phenols, was around 3400 cm^−1^. The spectrum of the L extract no longer showed peaks at 2860.12 and 1744.65 cm^−1^, while that of the L-AgNPs showed peaks at 577.39 and 526.03 cm^−1^. A significant change from 477.98 cm^−1^ in the L extract to 414.44 cm^−1^ in the spectra of the L-AgNPs was also observed. The -CH stretching of the alkyl group may be responsible for the peaks at 2927 and 2860 cm^−1^, the -enolic diketones or carboxylic acids -C=O pulling the peak at 1651 cm^−1^, and the corresponding -CO-O stretching at 1375 and 1022 cm^−1^. These peaks in the L-AgNPs indicate that the nanoparticles were immobilized and coated with phytochemicals present in the *S. costus* leaves, including flavonoids, alkaloids, phenols, and organic acids. The results confirm previous research findings [58]. FTIR analysis confirmed that the nanoparticles were strongly coated with many bioactive organic components present in the *S. costus* leaf extract, resulting in lower peak intensities of the bands seen in AgNPs, suggesting that the biomolecules may have been adsorbed on the surface of the metal ions after interacting with their oxygen-donating atoms [61]. Similarly, for the R-AgNPs, capping and stabilization are attributed to specific functional groups, which were identified by comparing the FTIR spectra of the R-AgNPs and R extract according to their peak positions. We recorded the infrared spectra of *S. costus* and its synthesis (Figure 8B). The AgNPs spectra showed a change in the broadened peak from 3418.15 cm^−1^ in the extract to 3409.68 cm^−1^. The expansion frequency of plant polyphenols, including hydrogen bonds, is responsible for this phenomenon. The presence of functional groups derived from seed extracts is suggested by the shift in the peaks observed in the formulation [62]. Likewise, the vibrations changed from 1034.79 to 1033.06 cm^−1^, which corresponds to the CO₂ stretching of the ether bonds due to flavones adsorbed on the surface of biotinylated AgNPs. It is suggested that the binding processes of the AgNPs with the extract are responsible for the disappearance of the peaks at 1743.23 cm^−1^, 1333.18 cm^−1^, and 1260.99 cm^−1^ in the *S. costus* root extract when these nanoparticles were included. The IR peaks at 1626.44 cm^−1^ and 1626.09 cm^−1^ indicate that the extract and AgNPs contain C=O and C=C bonds or aromatic C=C bonds, respectively. The vibration peaks at 1034.79 and 1033.06 cm^−1^ are associated with carbon dioxide stretching. During the green synthesis of AgNPs, there was a noticeable change in the FTIR spectra of the double and triple hydrocarbon bonds and the OH- or N-H-stretching peaks [63]. In addition, new peaks at 934.11, 873.39, and 778.03 cm^−1^ indicate the possibility of attaching CH groups to AgNPs.

### 2.2. Biological Studies

#### 2.2.1. Antioxidant Activity of AgNPs

**Figure 9:**
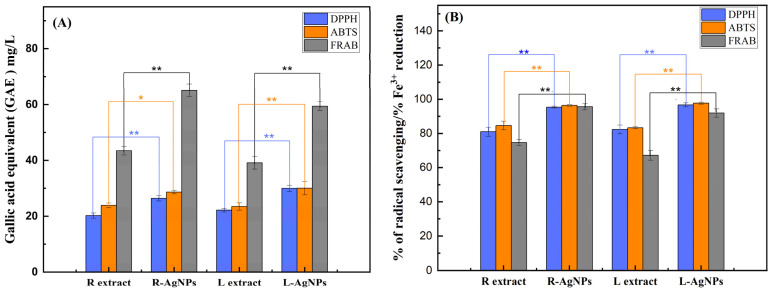
Antioxidant activity of AgNPs synthesized from *S. costus* extracts and determined by ABTS, DPPH, and FRAP methods. Results presented as the mean ± SD (*n* = 3) are expressed as GAE in mg/L (**A**) and as % of radical scavenging or as % of Fe^3+^ reduction in both ABTS and DPPH methods or in the FRAP method (**B**). L extract: aqueous leaves extract; R extract: aqueous roots extract; L-AgNPs: AgNPs prepared using L extract; R-AgNPs: AgNPs prepared using R extract. The asterisk indicates significant differences between experimental groups within a column. * represents *p* < 0.05; ** represents *p* < 0.01.

ABTS, DPPH, and FRAP methods were used to investigate the antioxidant activities of both root and leaf aqueous extracts from *S. costus* as well as their respective silver nanoparticles (AgNPs) prepared by green synthesis. The recorded results shown in Figure 9 are expressed as gallic acid equivalent (GAE) in mg/L (Figure 9A) and as percentage of radical scavenging or as percentage of Fe^3+^ reduction in both ABTS and DPPH methods or in FRAP method (Figure 9B), respectively.

AgNPs prepared using root and leaf extracts showed higher significant antioxidant activity compared with their respective aqueous extracts, while no activity was recorded for the AgNO_3_ solution.

Indeed, using the ABTS and DPPH methods, recorded data ranged between 81–84.7% or 95.3–97.7% of radical scavenging and 20.3–23.9 or 26.4–30.7 GAE mg/L for aqueous extracts or AgNPs, respectively. The highest antioxidant activity was determined in the R-AgNPs (95.6 ± 1.5% of Fe^3+^ reduction; 65.1 ± 2.3 GAE mg/L) when using the FRAP method. Similarly, a significantly higher antioxidant activity was observed with the L-AgNPs (91.9 ± 2.5% of Fe^3+^ reduction; 43.5 ± 1.5 GAE mg/L) compared with the aqueous leaf (74.7 ± 1.7% of Fe^3+^ reduction; 59.5 ± 1.6 GAE mg/L) and root (67.2 ± 2.8% of Fe^3+^ reduction; 39.2 ± 2.6 GAE mg/L) extracts. This antioxidant activity could be attributed to the presence of phenolic compounds in aqueous extracts of *S. costus* as well as on the surface of the nanoparticles. These compounds act as capping and antioxidant agents by providing electrons to metals and free radicals, and thereby help their stabilization. In fact, the ease with which a hydroxyl group is given to a free radical and the aromatic structure’s capacity to hold an unpaired electron are the main causes of several polyphenols’ antioxidant activities [64]. The current findings could be supported by previous works demonstrating the abundance in *S. costus* of phenolic compounds in addition to flavonoids and essential oils [65]. ScAEs’ polyphenols therefore exhibited a significant impact as AgNPs capping agents as well as silver ion reducing agents.

#### 2.2.2. Enzyme Inhibition of AgNPs

**Figure 10:**
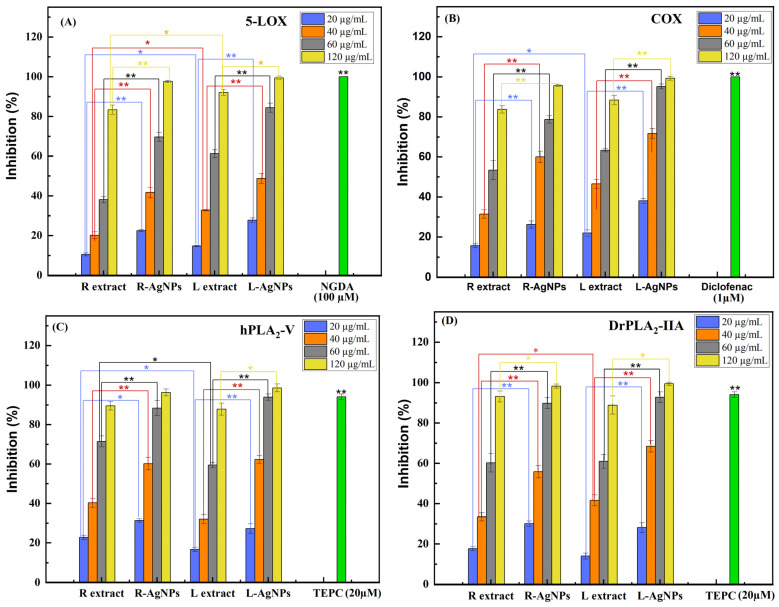
Evaluation of 5-LOX, COX, and sPLA_2_ inhibition properties of AgNPs synthesized from *S. costus* extracts. Several concentrations ranging from 20 to 120 µg/mL of *S. costus* root and leaf extracts and their silver nanoparticles AgR and AgL were used to inhibit 5-LOX (**A**), COX (**B**), h-PLA_2_-V (**C**), and DrPLA_2_-IIA (**D**). NGDA at 100 µM, diclofenac at 1 µM, and TEPC at 20 µM served as positive controls. Results are presented as the mean ± SD (*n* = 3). L extract: aqueous leaves extract; R extract: aqueous roots extract; L-AgNPs: AgNPs prepared using L extract; R-AgNPs: AgNPs prepared using R extract. * represents *p* < 0.05; ** represents *p* < 0.01.

The in vitro anti-inflammatory activity of the root and leaf extracts compared with their respective AgNPs was evaluated by determining the inhibition percentage of enzymes involved in inflammation, such as COX-1, 5-LOX, and sPLA_2_s (hPLA_2_-V and DrPLA_2_-IIA). Aqueous extracts and synthesized AgNPs were tested at concentrations ranging from 20 to 120 µg/mL. The data presented in Figure 10 show clearly that the AgNPs displayed significant in vitro anti-inflammatory potency comparable to that of the standard drug used as the positive control. Indeed, AgNPs exhibited a better enzymatic inhibitory effect, in a dose-dependent manner, at concentrations of 20, 40, 60, and 120 μg/mL toward all the tested enzymes than the aqueous root and leaf extracts (Figure 10). The highest enzymatic inhibition was observed at 120 μg/mL, showing that AgNPs were more effective against 5-LOX (Figure 10A), COX-1 (Figure 10B), hPLA_2_-V (Figure 10C), and DrPLA_2_-IIA (Figure 10D) (of about 99% inhibition) than aqueous root and leaf extracts (of about 85% inhibition). At 120 µg/mL, the AgNPs suppressed COX and 5-LOX activity to a degree comparable to when diclofenac (1 µM) and NGDA (100 µM) were used as positive controls, respectively (Figure 10A,B). R-AgNPs and L-AgNPs inhibited hPLA_2_-V and DrPLA_2_-IIA up to 97%, while TEPC (20 µM) caused 94% inhibition of both PLA_2_ (Figure 10C,D). 

These promising results for AgNPs synthesized from *S. costus* root and leaf extracts were in line with previous findings of several medicinal plants’ extracts and pure compounds that demonstrated significant anti-inflammatory properties and potential development of modern anti-inflammatory drugs [66]. Moreover, it was reported that extracts of *Heritiera fomes* and its AgNPs revealed significant in vitro anti-inflammatory activity [67]. A high reduction in pro-inflammatory cytokine levels was induced by the aqueous extract of *Cotyledon orbiculata*-based AgNPs [68]. AgNPs synthesized using extracts of *Naphtha* spp. displayed significant inhibitory efficiency against COX-1 and COX-2 enzymes compared with standard drugs [69]. Costunolide, cynaropicrin, and santa marin isolated from *Saussurea lappa* extract proved their anti-inflammatory activity by inhibiting the production of inflammatory factors and lymphocytes’ proliferation and blocking inducible nitric oxide synthase (iNOS) protein, suppressing COX-2 and COX-derived prostaglandin E2 production in LPS-stimulated RAW264.7 cells and murine peritoneal macrophages [70].

The current results were very interesting for designing new cancer drugs since arachidonic acid (AA) signaling is a critical inflammatory pathway that is upregulated in most cancers and causes drug resistance [71]. Thus, enzymes involved in inflammation mediator production represent an attractive target for cancers in which inflammation plays an important role. Indeed, in AA signaling pathways, sPLA_2_s are the key enzymes that ensure the release of AA from membrane phospholipids. AA is further metabolized by downstream enzymes (COX/LOX) in order to produce eicosanoids, known as inflammatory lipid mediators. Therefore, LOX and COX pathway-derived eicosanoids play a critical role during the inflammation process [72]. Hence, inhibition of enzymes involved in eicosanoids’ production is a promoting strategy in controlling chronic inflammation in several cancers.

The inhibition of COX-1, 5-LOX, and sPLA_2_ enzymes by *S. costus* silver nanoparticles for anti-inflammatory study could be considered a novelty that certainly opens several perspectives for their therapeutic investigation for several diseases.

#### 2.2.3. Anti-Tumoral Effect of AgNPs

**Figure 11:**
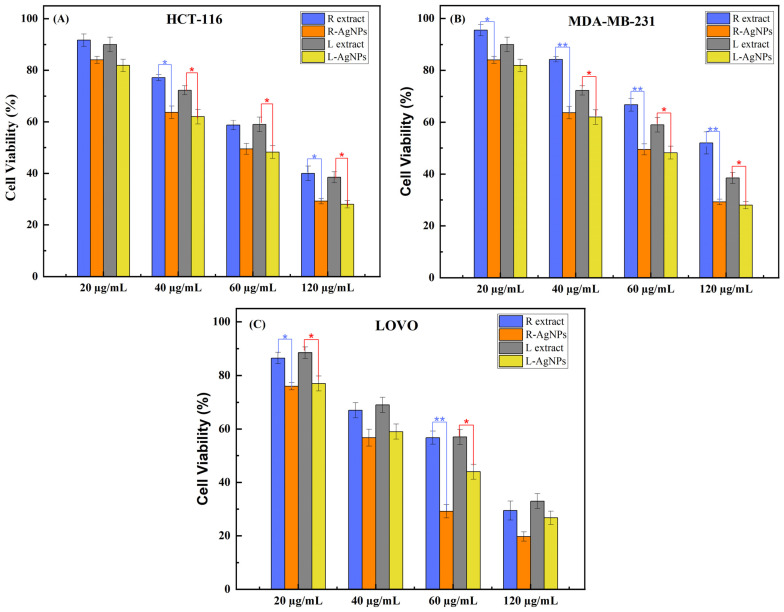
Cytotoxic potential of AgNPs synthesized from *S. costus* extracts against human colorectal HCT-116 (**A**), LoVo (**B**), and breast cells MDA-MB-231 (**C**). Cells were treated with several concentrations of extracts and silver nanoparticles during 24 h, from 20 µg/mL to 120 µg/mL. MTT assay was used to quantify viable cells. Current results are presented as the mean ± SD (*n* = 3 assays). L extract: aqueous leaves extract; R extract: aqueous roots extract; L-AgNPs: AgNPs prepared using L extract; R-AgNPs: AgNPs prepared using R extract. The asterisk indicates significant differences between experimental groups within a column. * represents *p* < 0.05; ** represents *p* < 0.01.

Three human cancer cell lines, HCT-116, LoVo, and MDA-MB-231, were used in order to evaluate the anti-tumoral effect of the AgNPs prepared from *S. costus* extracts. Therefore, MTT assay was assessed by treating cells with several concentrations of each extract and nanoparticles from 20 to 120 µg/mL. The data represented in Figure 11 demonstrated that the root/leaf aqueous extracts, as well as the synthesized AgNPs, inhibited the proliferation of human cancer cells in a dose-dependent manner. A highly significant decrease in HCT-116, LoVo, and MDA-MB-231 cells (more than 70%) was observed at 120 µg/mL of AgNPs. L-AgNPs and R-AgNPs exhibited IC_50_ values of 56 µg/mL and 60 µg/mL toward HCT-116 and MDA-MB-231, respectively (Table 1), while IC_50_ values of 50 µg/mL and 42 µg/mL were recorded toward LoVo cells for L-AgNPs and R-AgNPs, respectively. However, the root and leaf aqueous extracts were less effective, showing IC_50_ values of 82 and 74 µg/mL against HCT-116 and MDA-MB-231, respectively. An IC_50_ value of 86 µg/mL was observed for the leaves extract toward MDA-MB-231. Similar results were described with *Saussurea lappa* genus extracts, which represent the most studied genus compared with *S. costus.* Moreover, regarding the anti-tumoral effect of the *Saussurea* genus, *Saussurea lappa* extracts are the most studied, and several compounds have been purified from these extracts with an anti-tumoral effect, especially dehydrocostus lactone, costunolide, and cynaropicrin [70]. Indeed, the chloroformic extract of *S. lappa* was cytotoxic against human breast cancer MDA-MB cells with a comparable effect to the standard compound, doxorubicin [70]. The cytotoxic mechanism of costunolide extracted from *S. lappa* toward MDA-MB-231 was determined and showed a metastatic suppression through inhibition of tumor necrosis factor alpha (TNF-α) as well as in vitro TNF-α-induced invasion and migration of MDA-MB-231 cells [73]. Cynaropicrin purified from *Saussurea lappa* extract repressed Jukart T, Eol-1, and U937 cell lines in a dose-dependent manner with IC_50_ values of 2.36, 10.90, and 3.11 µmol/L, respectively. Dehydrocostus lactone from *Saussurea lappa* exhibited high hepatocellular carcinoma activity [70]. The water extract of *S. lappa* inhibited the proliferation and invasion of intestinal cancer cell lines, probably due to the presence of costunolide [70]. Synthesized zinc oxide nanoparticles (ZnONPs) using *Saussurea lappa* root were cytotoxic at 5 μg/mL on the CHO cell line with an IC_50_ value of 3.164 µg/mL [35]. Compared with *Saussurea lappa* extracts, few studies have described the anti-tumoral effect of *S. costus* extracts or their synthetized nanoparticles. MgONPs synthetized with root extracts of two varieties of *S. costus* collected from two regions of Pakistan induced MCF-7 cells’ cytotoxicity in a dose-dependent manner with IC_50_ values of 67.3% and 52.1%. This difference in cytotoxicity was related to the concentration of active constituents in the two varieties of *S. costus*. MgONPs caused cell damage, especially rounding, shrinking, and membrane blebbing as well as chromatin condensation of the treated MCF-7 cells. 

The mechanism of the anti-tumoral effect of MgONPs was investigated and showed a high apoptosis induction with activation of intrinsic pathways due to loss of mitochondrial membrane potential and ROS accumulation [27]. Generated palladium nanoparticles (PdNPs) from *S. costus* extract reduced the viability of colon cancer (HCT-116), hepatocellular carcinoma (HepG2), and breast adenocarcinoma (MCF-7) cell lines [74].

#### 2.2.4. Antimicrobial Effect of AgNPs

The antibacterial activity of AgNPs synthesized from *S. costus* extracts was found by determining the colony-forming ability (CFU) of bacteria or fungi strains incubated with various concentrations of aqueous extracts or AgNPs from 20 to 120 µg/mL. Two Gram-positive bacteria, *Staphylococcus aureus* (ATCC 25923) and *Enterococcus faecalis* (ATCC 29122), and two Gram-negative bacteria, *Bacteroides fragilis* (ATCC 25285) and *E. coli* (ATCC 25922), were used. The bactericidal activity of *S. costus* extracts and AgNPs was evaluated as the residual CFU value compared with that of the initial inoculum. The IC_50_ value corresponds to the concentration of the tested extracts or AgNPs that inhibited the growth of 50% of the initial inoculum. The positive control was the antibiotic ampicillin. Table 2 clearly shows that AgNPs synthesized from *S. costus* extracts were more effective than *S. costus* root and leaf extracts as well as ampicillin against all bacterial strains, with IC_50_ values ranging from 14 to 20 µg/mL. In addition, the Gram-positive strains *Enterococcus faecalis* and *Staphylococcus aureus* seemed to be more sensitive to the nanoparticles L-AgNPs, with IC_50_ values of 14.3 and 16.25 µg/mL, respectively. According to Al-Saggaf et al., 2020, bioactive constituents identified and purified from *S. costus* extract with strong antimicrobial potentiality were mostly terpenoids, glycosides, flavonoids, and lactones [31].

Different solvent extracts (methanolic, ethanolic, aqueous, petroleum ether) of *Saussurea lappa* have been tested and found to be effective against a variety of resistant pathogens [70]. However, few studies have described the antimicrobial effect of *Saussurea* nanoparticles. The phytosynthesized SCE/Se-NP generated using *S. costus* root extract (SCE) and selenium (Se) was more effective against Gram (−) and Gram (+) bacterial strains than SCE with MIC 17.5, 20, and 25 µg/mL toward *S. typhimurium*, *E. coli,* and *S. aureus*, respectively. The examination of the morphology and deformation of treated bacterial cells revealed that SCE/Se-NP appeared attaching to the cell walls, resulting in their lysis [31]. MgONPs synthetized with root extracts of two varieties of *S. costus* from two regions of Pakistan displayed potent antibacterial activity in a dose-dependent manner against *E. coli*, *P. aeruginosa*, *S. aureus,* and *B. subtilis*. The bactericidal effect of the MgONPs, involving cell and membrane damage, was attributed to oxidative stress induced by the spontaneous release of ROS and RNS free radicals or electrochemical interactions between LPS and Mg^2+^ ions in the nanoparticles. The growth inhibition of the bacterial strains was related to the penetration of nanoparticles into the bacterial cell, causing damage, releasing the cell contents, and consequently leading to cell death [27]. At 170 ppm, zinc oxide nanoparticles (ZnONPs) using *Saussurea lappa* root exhibited a better antimicrobial effect against Gram-negative strains (*Sphingobacterium thalpophilum*, *Staphylococcus aureus*, *E. coli*, *Pseudomonas aeruginosa*, *Sphingobacterium* sp., *Acinetobacter* sp., *Ochrobactrum* sp.) than Gram-positive strains (*Streptococcus aureus*, *Bacillus subtilis*) compared with concentrations of 50 and 100 ppm. This effect was related to the structure of the cell membrane, cell physiology and metabolism, and degree of contact with the nanoparticles [35]. AgNPs synthetized from *S. lappa* roots exhibited significant antibacterial potential toward *Bacillus cereus* and *E. coli* strains [75].

The antifungal activity of AgNPs synthesized from *S. costus* extracts was evaluated by the disc diffusion method on Sabouraud dextrose agar against three fungal strains, *Penicillium digitatum*, *Aspergillus niger*, and *Aspergillus oryzae,* and further compared with cycloheximide at 1 mg/mL, which served as positive control. It is easy to see, from Table 2, the effectiveness of the AgNPs against all tested fungi strains, which was twofold higher than that of their respective aqueous extracts. However, the recorded IC_50_ values were still lower than those of the standard drug, cycloheximide. Moreover, Table 2 shows that *A. oryzae* was more sensitive to AgNPs as well as both aqueous extracts. This antifungal activity could be attributed to the nanoparticles’ interaction with the fungal strains’ outer membranes, resulting in structural modification, degradation, and ultimately, cell lysis. The inhibition of fungal growth might also be due to the release of ROS (reactive oxygen species), destabilization of DNA replication upon treatment with the metal ion nanoparticles and ribosomal expression, as well as the inactivation of enzymes involved in ATP production [35]. Few studies have reported the antifungal activity of *Saussurea* nanoparticles. Indeed, the NCt/CE nanoconjugates generated from the biopolymer (chitosan) and *S. costus* root extract had significantly high antimicrobial activity against antibiotic-resistant strains from *C. albicans* and *C. glabrata,* with vigorous structural and morphological alterations [76]. The antifungal activity of ZnONPs, at 170 ppm, toward *Aspergillus niger* and *Aspergillus flavus*, *Fusarium oxysporum,* and *Rhizopus* strains was attributed to higher interactions between positively charged nanoparticles and negatively charged microorganisms’ cell membranes compared with crude extract and was facilitated by their chemical stability, surface chemistry, and smaller size [35]. Collectively, *Saussurea*-generated nanoparticles could be a potent natural, biodegradable, and effective antifungal and antibacterial agent to control resistant pathogenic strains.

## 3. Material and Methods

### 3.1. Green Synthesis and Characterization of AgNPs

The *S. costus* plant leaves (L) and roots (R) were collected from a Qassim, Saudi Arabia local market. The collected plant was washed well, dried, and ground to form a powder. The powdered leaves and roots (10 g) were macerated in 100 mL of boiling distilled water separately and left overnight. After filtering, 5 milliliters of the L and R aqueous extracts were combined with about 50 milliliters of 1 millimolar silver nitrate separately. The silver nitrate was not photoactivated throughout the reaction; therefore, it was conducted in total darkness. The reaction mixture was stirred continuously at 45 °C to start producing nanoparticles. After 20 to 30 min, the reduction of AgNO_3_ to AgNPs was visible, and the distinctive dark brown color identified the reaction [10] (Figure 1).

The optical properties of the synthesized silver nanoparticles (AgNPs) were evaluated using UV–Vis spectroscopy (Shimadzu-1800, Kyoto, Japan) and a Fluorolog 3 fluorescence spectrometer (Nytek Instruments JSC, Moscow Region, Russia). The average size of the AgNPs was determined using dynamic light scattering (DLS) combined with a Zetasizer from Malvern Instruments (Worcestershire, United Kingdom), specifically, the HT Laser, ZEN3600 Malvern, Nano series. Fourier transform infrared (FTIR) spectroscopy (Shimadzu IR, Prestige 21, Nakagyo-Ku, Japan) was employed to distinguish between the biomolecule functional groups present in the *S. costus* extract and those in the synthesized AgNPs. Transmission electron microscopy (TEM; JEM-1400; JEOL; Tokyo, Japan) was used to examine the surface appearance and size distributions of the produced nanoparticles. Energy dispersive X-ray spectroscopy (EDX) (JEM-2100F TEM; JEOL; Tokyo, Japan) confirmed the presence of various elements and components in the suspension. For X-ray diffraction (XRD) analysis, a D8 Advance X-ray diffractometer (Bruker, Billerica, MA, USA) was utilized in conjunction with a graphite monochromator and a CuKα source (λ = 1.5418 Å) operating at 45 kV and 40 mA.

### 3.2. Antioxidant Activity

Investigation of the antioxidant potency of the extracts was performed using the 2,2 diphenyl-1-picrylhydrazyl (DPPH) radical scavenging technique based on the stable DPPH free radical’s capacity to interact with hydrogen donors [77]. Briefly, the absorbance of the mixture of each sample (15 µL) and 0.095 mM DPPH (150 µL) was measured for 12 min at 505 nm. Furthermore, the free radicals’ concentration was spectrophotometrically determined using the common 2,2′-azino-bis (3-ethylbenzothiazoline-6-sulfonic acid) (ABTS) radical scavenging method, which is based on the neutralization of a radical cation produced by the synthetic chromophore ABTS’s one-electron oxidation. Each sample (3 µL) was incubated with 7 mM ABTS (150 µL) for 12 min, and the absorbance at 660 nm was measured. The ferric reducing antioxidant power (FRAP) method was also carried out to evaluate the antioxidant activity of the four tested extracts. This is based on the 2,4,6-tripyridyl-s-triazine (TPTZ) reduction with ferric chloride hexahydrate (FeCl_3_·6H_2_O) forming blue ferrous complexes. First, three solutions of 10 mM TPTZ (prepared in 40 mM hydrochloric acid), 20 mM FeCl_3_, and 20 mM acetate buffer (pH 3.6) were mixed in a 1:1:10 ratio. Then, each sample (3 μL) was pipetted into a plastic cuvette containing the prepared reagent (150 μL), and the absorbance was followed for 12 min at 605 nm. The difference in the absorbance at the appropriate wavelength between the 12th and 2nd minute of each assay procedure was used to determine the antioxidant activity.

### 3.3. Antimicrobial Activity

Pure standard bacterial isolates collected from King Khaled University Hospital, including *Staphylococcus aureus* (ATCC 25923) and *Enterococcus faecalis* (ATCC 29122) as Gram-positive bacteria, and *Bacteroides fragilis* (ATCC 25285) and *Escherichia coli* (ATCC 25922) as Gram-negative bacteria, were tested in this study. For bacterial suspension preparation of 0.5 MacFarland, small inoculums from fresh cultures of each microorganism grown on nutrient agar plates were suspended in nutrient broth (5 mL). Bacterial viability was examined by determining colony-forming ability (CFU) of bacteria incubated without or with different amounts (0–100 μg/mL) of each extract that was mixed with 2 × 10^7^ CFU/mL in sterile brain heart infusion (BHI) broth and incubated for 1 h under shaking at 37 °C. Serially diluted samples in sterile BHI were streaked onto media agar plates and incubated for 24 h at 37 °C. The antibacterial potency of each investigated extract was expressed as the CFU residual number with reference to the initial inoculums, and the IC_50_ (the concentration necessary to kill 50% of the initial inoculum) values were deduced from curves obtained from two independent experiments. Ampicillin (1 mg/mL) was used as a standard drug.

Evaluation of the antifungal activity of studied extracts on three fungal strains (*Penicillium digitatum*, *Aspergillus niger*, *Aspergillus oryzae*) was performed by disc diffusion technique using Sabouraud dextrose agar [78]. Each extract (10 µL) of the standard drug cycloheximide (1 mg/mL) was placed on sterile paper discs that were subsequently deposited in the middle of the inoculated Petri dishes and kept for 24 h at 30 °C.

### 3.4. Cell Culture

Human colon (HCT-116 and Lovo) and breast (MDA-MB-231) cancer cell lines (American Type Culture Collection; Manassas, VA, USA) were used in the current study to investigate the cytotoxic potency of aqueous extracts and AgNPs. The MTT assay was used to evaluate the metabolic activity of cells that were grown in a 5% CO_2_-humidified incubator in Dulbecco’s Modified Eagle’s Medium supplemented with fetal bovine serum (15%) for 24 h at 37 °C. In a 96-well plate, different amounts (0–120 µg) of each sample, previously diluted in culture medium, were incubated with cells (4 × 10^4^ in each well) at 37 °C for 24 h. A subsequent incubation for 4 h was performed after adding 20 µL of MTT (5 mg/mL in PBS) to the cells. Then, the medium was removed and replaced with an equal volume of saline solution. Finally, the preparations were well mixed using a shaker to dissolve the formazan crystals. Cell viability was expressed as a relative percentage of the OD values recorded at 550 nm for treated cells relative to the control (without any extract or AgNPs). The plot of the cell viability (%) versus the extract concentration was performed to determine the extract concentration providing 50% inhibition (IC_50_).

### 3.5. Cyclooxygenase and Lipoxygenase Inhibition Assays

Each sample was investigated in duplicates at 20, 40, 60, and 100 μg/mL using commercial COX and LOX inhibitory screening assay kits (catalog numbers: 560131 and 766700, respectively; Cayman Chemical Company Ann Arbor, MI, USA) following the manufacturer’s instructions. Diclofenac (1 µM) and nordihydroguaiaretic acid (100 µM) were used as the standard drugs for inhibition of COX-1/2 and 5-LOX, respectively.

### 3.6. Inhibition of sPLA_2_ Activity

The inhibitory potency of aqueous extracts and AgNPs was assayed using human group V (hPLA_2_-V) and dromedary group-IIA (DrPLA_2_-IIA) sPLA_2_ (0.02 μg/μL) according to the De Aranjo and Radvany (1987) protocol [79]. A mixture of each sample (20, 40, 60, and 100 μg/mL) and each sPLA_2_ (10 μL) was first incubated at room temperature for 20 min. Then, PLA_2_ substrate (1 mL of 3.5 mM lecithin solubilized in 100 mM NaCl, 10 mM CaCl_2_, 3 mM NaTDC, and 0.055 mM red phenol, pH 7.6) was added and the hydrolysis reactions were followed spectrophotometrically for 5 min at 558 nm. The inhibition percentage was calculated by comparison with a control (absence of any extract or AgNPs). The IC_50_ values were deduced from the plotted curves. All measurements were in duplicate.

### 3.7. Statistical Analysis

Data were statistically analyzed using SPSS software (ver.22; SPSS Inc., Chicago, IL, USA). Group differences were studied using Tukey’s post hoc test. The limit of the significance of all analyses was considered significant at a value of *p* < 0.05 (*) and *p* < 0.01 (**).

## 4. Conclusions

With the help of a green, eco-friendly, simple, and quick method for creating nanoparticles, *Saussurea costus* root and leaf extract was successfully used to create silver nanoparticles, or AgNPs. To our knowledge, the comparative study of silver nanoparticles from *S. costus* leaves and roots and their biological effects has never been performed in previous studies, and the current work could be considered to be pioneering in this idea.

AgNP biosynthesis is a safe substitute for potentially dangerous physical and chemical processes. This study explored the physiochemical attributes of L-AgNP and R-AgNP extracts using various analytical methods. UV spectroscopy confirmed the presence of silver nanoparticles by detecting peaks within the 400–500 nm range. TEM imaging also supported the generation of silver nanoparticles consistent with the UV findings and emphasizing surface plasmon resonance effects. Fluorescence spectroscopy revealed a shift toward the blue end in the emission peak as nanoparticle size decreased, suggesting heightened interactions. L-AgNPs had an average size of 148.8 nm, with R-AgNPs being smaller, at 50.32 nm. Both types demonstrated uniform distribution and stability, as evidenced by their polydispersity index values. Interestingly, the synthesized AgNPs had a synergistic impact as anti-inflammatory, anti-tumoral, and antibacterial biomolecules. Nevertheless, while the AgNPs showed promising applications in various fields, their use in in vitro studies may not accurately mimic the distribution and exposure patterns and therefore can significantly impact the reliability and generalizability of the current findings. Consequently, further in vivo studies, such as exposure of animals to synthesized AgNPs, are needed, which could help assess systemic effects, biodistribution, and potential long-term toxicity.

## Figures and Tables

**Table 1:** IC_50_ values of AgNPs synthesized from *S. costus* extracts against human colorectal HCT-116 and LoVo cell lines and breast cells MDA-MB-231.

	IC_50_ (µg/mL)		
	HCT-116	LoVo	MDA-MB-231
R extract	82	74	120
L extract			86
R-AgNPs	60	42	60
L-AgNPs	56	50	56

L extract: aqueous leaves extract; R extract: aqueous roots extract; L-AgNPs: AgNPs prepared using L extract; R-AgNPs: AgNPs prepared using R extract.

**Table 2:** Antimicrobial activities of AgNPs synthesized from *S. costus* extracts against Gram (+) and Gram (−) bacterial and fungal strains. IC_50_ value, which was deduced from curves obtained from two independent experiments, corresponds to the concentration of tested extracts or AgNPs that inhibited the growth of 50% of the initial inoculum. Results are presented as the mean ± SD (*n* = 3).

Pathogens	IC_50_ (μg/mL)				
Bacteria	**R Extract**	**L Extract**	**R-AgNPs**	**L-AgNPs**	**Ampicillin**
*B. fragilis* (ATCC 25285)	24.5 ± 0.98	29.25 ± 1.76	17.85 ± 1.2	17.55 ± 2.19	16.15 ± 1.2
*E. coli* (ATCC 25922)	29.7 ± 1.83	26.9 ± 2.26	20.65 ± 2.33	14.3 ± 0.98	20.25 ± 1.76
*E. faecalis* (ATCC 29122)	21.65 ± 0.91	24.45 ± 1.34	14.8 ± 0.98	14.3 ± 1.83	12.75 ± 1.06
*S. aureus* (ATCC 25923)	21 ± 2.82	23.5 ± 2.12	16 ± 1.41	16.25 ± 1.06	17 ± 1.41
Fungi	**R Extract**	**L Extract**	**R-AgNPs**	**L-AgNPs**	**Cycloheximide**
*A. niger*	10.25 ± 0.63	11.5 ± 0.42	5.3 ± 0.28	5.85 ± 0.63	2.65 ± 0.21
*P. digitatum*	12.8 ± 0.98	9.35 ± 0.21	7.2 ± 0.56	4.9 ± 0.42	3.15 ± 0.21
*A. oryzae*	3.75 ± 0.35	4.625 ± 0.53	2.5 ± 0.70	3.25 ± 0.35	2.5 ± 0.35

L extract: aqueous leaves extract; R extract: aqueous roots extract; L-AgNPs: AgNPs prepared using L extract; R-AgNPs: AgNPs prepared using R extract.

## Data Availability

All data are available in the main text.

## References

[1] Sastry M., Ahmad A., Khan M.I., Kumar R., Niemeyer C.M., Mirkin C.A. (2004). Microbial nanoparticle production. Nanobiotechnology.

[2] Bhattacharya D., Rajinder G. (2005). Nanotechnology and potential of microorganisms. Crit. Rev. Biotechnol..

[3] Mohanpuria P., Rana N.K., Yadav S.K. (2008). Biosynthesis of nanoparticles: Technological concepts and future applications. J. Nanopart Res..

[4] Divya T., RAJ Yamuna K., Ayisha S., Joseph P. (2010). Synthesis of silver phyto nanoparticles and their antibacterial efficacy. Dig. J. Nanomat. Biostruct..

[5] Karim N., Liu S., Rashwan A.K., Xie J., Mo J., Osman A.I., Rooney D.W., Chen W. (2023). Green synthesis of nanolipo-fbersomes using Nutriose^®^ FB 06 for delphinidin-3-O-sambubioside delivery: Characterization, physicochemical properties, and application. Int. J. Biol. Macromol..

[6] Xu Y., Rashwan A.K., Osman A.I., Abd El-Monaem E.M., Elgarahy A.M., Eltaweil A.S., Omar M., Li Y., Mehanni A.-H.E., Chen W. (2023). Synthesis and potential applications of cyclodextrinbased metal-organic frameworks: A review. Environ. Chem. Lett..

[7] Monga Y., Kumar P., Sharma R.K., Filip J., Varma R.S., Zbořil R., Gawande M.B. (2020). Sustainable synthesis of nanoscale zerovalent iron particles for environmental remediation. ChemSusChem.

[8] Rashwan A.K., Karim N., Xu Y., Hanafy N.A.N., Li B., Mehanni A.-H.E., Taha E.M., Chen W. (2023). An updated and comprehensive review on the potential health effects of curcumin-encapsulated micro/nanoparticles. Crit. Rev. Food Sci. Nutr..

[9] Osman A.I., Zhang Y., Farghali M., Rashwan A.K., Eltaweil A.S., Abd El-Monaem E.M., Mohamed I.M.A., Badr M.M., Ihara I., Rooney D.W. (2024). Synthesis of green nanoparticles for energy, biomedical, environmental, agricultural, and food applications: A review. Environ. Chem. Lett..

[10] Abd El-Aziz A.R.M., Gurusamy A., Alothman M.R., Shehata S.M., Hisham S.M., Alobathani A.A. (2021). Silver nanoparticles biosynthesis using *Saussurea costus* root aqueous extract and catalytic degradation efficacy of safranin dye. Saudi J. Biol. Sci..

[11] Rashwan A.K., Bai H., Osman A.I., Eltohamy K.M., Chen Z., Younis H.A., Al-Fatesh A., Rooney D.W., Yap P.-S. (2023). Recycling food and agriculture by-products to mitigate climate change: A review. Environ. Chem. Lett..

[12] Rashwan A.K., Karim N., Xu Y., Xie J., Cui H., Mozafari M.R., Chen W. (2023). Potential micro-/nano-encapsulation systems for improving stability and bioavailability of anthocyanins: An updated review. Crit. Rev. Food. Sci. Nutr..

[13] Fang C., Ma Z., Chen L., Li H., Jiang C., Zhang W. (2019). Biosynthesis of gold nanoparticles, characterization and their loading with zonisamide as a novel drug delivery system for the treatment of acute spinal cord injury. J. Photochem. Photobiol. B.

[14] Owoseni-Fagbenro K.A., Saifullahm S., Imran M., Perveen S., Rao K., Fasina T.M., Olasupo I.A., Adams L.A., Ali I., Shah M.R. (2019). Egg proteins stabilized green silver nanoparticles as delivery system for hesperidin enhanced bactericidal potential against resistant *S. aureus*. J. Drug. Deliv. Sci. Technol..

[15] Devanesan S., AlSalhi M.S. (2021). Green synthesis of silver nanoparticles using the flower extract of *Abelmoschus esculentus* for cytotoxicity and antimicrobial studies. Int. J. Nanomed..

[16] Yusefi M., Shameli K., Yee O.S., Teow S.-Y., Hedayatnasab Z., Jahangirian H., Webster T.J., Kuča K. (2021). Green synthesis of Fe_3_O_4_ nanoparticles stabilized by a *Garcinia mangostana* fruit peel extract for hyperthermia and anticancer activities. Int. J. Nanomed..

[17] Al-Olayan E., Almushawah J., Alrsheed H., Dawoud T.M., Abdel-Gaber R. (2023). Potential role of biosynthesized silver nanoparticles from *Aaronsohnia factorovskyi* on Hymenolepis nana in BALB/c mice. Arq. Bras. Med. Vet. Zootec..

[18] Bruna T., Maldonado-Bravo F., Jara P., Caro N. (2021). Silver Nanoparticles and Their Antibacterial Applications. Int. J. Mol. Sci..

[19] Ashkar M.A., Babu A., Joseph R., Kutti Rani S., Vasimalai N. (2023). Ecofriendly synthesis of silver nanoparticles using Radish microgreens extract and their potential photocatalytic degradation of toxic crystal violet and pyronin Y dyes and antibacterial studies Inorg. Chem. Commun..

[20] Eswaran S.G., Narayan H., Vasimalai N. (2021). Reductive photocatalytic degradation of toxic aniline blue dye using green synthesized banyan aerial root extract derived silver nanoparticles. Biocatal. Agric. Biotechnol..

[21] Rafique M., Sadaf I., Rafique M.S., Tahir M.B. (2016). A review on green synthesis of silver nanoparticles and their applications. Artif. Cells Nanomed. Biotechnol..

[22] Jain A.S., Pawar P.S., Sarkar A., Junnuthula V., Dyawanapelly S. (2021). Bionanofactories for green synthesis of silver nanoparticles: Toward antimicrobial applications. Int. J. Mol. Sci..

[23] Ankita M., Ashish T., Srikanta M., Subhendu C., Susnata S.M., Arijit M., Suddhasattya D., Prakash C. (2024). Silver nanoparticle for biomedical applications: A review. Hybrid. Adv..

[24] AlMasoud N., Alomar T.S., Awad M.A., El-Tohamy M.F., Soliman D.A. (2020). Multifunctional green silver nanoparticles in pharmaceutical and biomedical applications. Green Chem. Lett. Rev..

[25] Abdallah E.M., Qureshi K.A., Ali A.M.H., Elhassan G.O. (2017). Evaluation of some biological properties of *Saussurea costus* crude root extract. Biosci. Biotech. Res. Comm..

[26] Pandey M.M., Rastogi S., Rawat A.K. (2007). *Saussurea costus*: Botanical, chemical and pharmacological review of an ayurvedic medicinal plant. J. Ethnopharmacol..

[27] Amina M., Al Musayeib N.M., Alarfaj N.A., El-Tohamy M.F., Oraby H.F., Al Hamoud G.A., Bukhari S.I., Moubayed N.M.S. (2020). Biogenic green synthesis of MgO nanoparticles using *Saussurea costus* biomasses for a comprehensive detection of their antimicrobial, cytotoxicity against MCF-7 breast cancer cells and photocatalysis potentials. PLoS ONE.

[28] Taha A. (2022). Microwave Sample Preparation System Assisted Biogenic Synthesis of Copper Oxide Nanoplates Using *Saussurea costus* Root Aqueous Extract and Its Environmental Catalytic Activity. Catalysts.

[29] Byambaragchaa M., de la Cruz J., Yang S.H., Hwang S.G. (2013). Anti-metastatic potential of ethanol extract of *Saussurea involucrata* against hepatic cancer in vitro. Asian Pac. J. Cancer Prev. APJCP.

[30] Mujammami M. (2020). Clinical significance of *Saussurea costus* in thyroid treatment. Saudi Med. J..

[31] Al-Saggaf M.S., Tayel A.A., Ghobashy M.O., Alotaibi M.A., Alghuthaymi M.A., Moussa S.H. (2020). Phytosynthesis of selenium nanoparticles using the *costus* extract for bactericidal application against foodbore pathogens. Green Process. Synth..

[32] Singh R., Chahal K.K., Singla N. (2017). Chemical composition and pharmacological activities of *Saussurea lappa*: A review. J. Pharmacogn. Phytochem..

[33] Mahapatra D.K., Tijare L.K., Gundimeda V., Nilesh M.M. (2018). Rapid Biosynthesis of Silver Nanoparticles of Flower-like Morphology from the root extract of *Saussurea lappa*. Res. Rev. A J. Pharmacogn..

[34] Aljohny B.O., Almaliki A.A.A., Anwar Y., Ul-Islam M., Kamal T. (2021). Antibacterial and Catalytic Performance of Green Synthesized Silver Nanoparticles Embedded in Crosslinked PVA Sheet. J. Polym. Environ..

[35] Kolahalam L.A., Prasad K.R.S., Murali Krishna P., Supraja N. (2021). *Saussurea lappa* plant rhizome extract-based zinc oxide nanoparticles: Synthesis, characterization and its antibacterial, antifungal activities and cytotoxic studies against Chinese Hamster Ovary (CHO) cell lines. Heliyon.

[36] Awad A., Alkhulaifi M., Aldosari S., Alzahly N.S., Aldalbahi A. (2019). Novel eco-synthesis of PD silver nanoparticles: Characterization, assessment of its antimicrobial and cytotoxicity properties. Materials.

[37] Dashora A., Rathore K., Raj S., Sharma K. (2022). Synthesis of silver nanoparticles employing Polyalthia longifolia leaf extract and their in vitro antifungal activity against phytopathogen. Biochem. Biophys. Rep..

[38] Manimaran K., Yanto D.H.Y., Anita S.H., Nurhayat O.D., Selvaraj K., Basavarajappa S., Kumarasamy K. (2023). Synthesis and characterization of Hypsizygus ulmarius extract-mediated silver nanoparticles (AgNPs) and test their potentiality on antimicrobial and anticancer effects. Environ. Res..

[39] Paosen S., Saising J., Septama A.W., Voravuthikunchai S.P. (2017). Green synthesis of silver nanoparticles using plants from Myrtaceae family and characterization of their antibacterial activity. Mater. Lett..

[40] Sharmin S., Islam M.B., Saha B.K., Ahmed F., Maitra B., Rasel M.Z.U., Rabbi M.A. (2023). Evaluation of antibacterial activity, in-vitro cytotoxicity, and catalytic activity of biologically synthesized silver nanoparticles using leaf extracts of Leea macrophylla. Heliyon.

[41] Malik M., Iqbal M.A., Malik M., Raza M.A., Shahid W., Choi J.R., Pham P.V. (2022). Biosynthesis and characterizations of silver nanoparticles from Annona squamosa leaf and fruit extracts for size-dependent biomedical applications. Nanomaterials.

[42] Shameli K., Ahmad M.B., Jazayeri S.D., Shabanzadeh P., Sangpour P., Jahangirian H., Gharayebi Y. (2012). Investigation of antibacterial properties of silver nanoparticles prepared via green method. Chem. Cent. J..

[43] Alaraidh I.A., Ibrahim M.M., El-Gaaly G.A. (2014). Evaluation of green synthesis of Ag nanoparticles using *Eruca sativa* and *Spinacia oleracea* leaf extracts and their antimicrobial activity. Iran. J. Biotechnol..

[44] Savithramma N., Rao M.L., Rukmini K., Devi P.S. (2011). Antimicrobial activity of silver nanoparticles synthesized by using medicinal plants. Int. J. ChemTech Res..

[45] Kulikouskaya V., Nikalaichuk V., Hileuskaya K., Ladutska A., Grigoryan K., Kozerozhets I., Sidarenka A. (2023). Alginate-coated biogenic silver nanoparticles for the treatment of Pseudomonas infections in rainbow trout. Int. J. Biol. Macromol..

[46] Ashraf J.M., Ansari M.A., Khan H.M., Alzohairy M.A., Choi I. (2016). Green synthesis of silver nanoparticles and characterization of their inhibitory effects on AGEs formation using biophysical techniques. Sci. Rep..

[47] Saion E., Gharibshahi E. (2011). On the theory of metal nanoparticles based on quantum mechanical calculation. Malays. J. Fundam. Appl. Sci..

[48] Paranga Z., Keshavarz A., Farahi S., Elahi S.M., Ghoranneviss M., Parhoodeh S. (2012). Fluorescence emission spectra of silver and silver/cobalt nanoparticles. Sci. Iran. Trans. F Nanotechnol..

[49] Ho N.T., Tien H.N., Jang S.J., Senthilkumar V., Park Y.C., Cho S., Kim Y.S. (2016). Enhancement of recombination process using silver and graphene quantum dot embedded intermediate layer for efficient organic tandem cells. Sci. Rep..

[50] Awad M.A., Hendi A.A., Ortashi K.M., Alzahrani B., Soliman D., Alanazi A., Alomar T.S. (2021). Biogenic synthesis of silver nanoparticles using *Trigonella foenum-graecum* seed extract: Characterization, photocatalytic and antibacterial activities. Sens. Actuators A Phys..

[51] Wang L., Wu Y., Xie J., Wu S., Wu Z. (2018). Characterization, antioxidant and antimicrobial activities of green synthesized silver nanoparticles from *Psidium guajava* L. leaf aqueous extracts. Mater. Sci. Eng. C.

[52] Rahimi-Nasrabadi M., Pourmortazavi S.M., Shandiz S.A.S., Ahmadi F., Batooli H. (2014). Green synthesis of silver nanoparticles using *Eucalyptus leucoxylon* leaves extract and evaluation of the antioxidant activities of the extract. Nat. Prod. Res..

[53] Labulo A.H., David O.A., Terna A.D. (2022). Green synthesis and characterization of silver nanoparticles using *Morinda lucida* leaf extract and evaluating its antioxidant and antimicrobial activity. Chem. Pap..

[54] Hendi A.A., Awad M.A., Alanazi M.M., Virk P., Alrowaily A.W., Bahlool T., Hagmusa B. (2023). Phytomediated synthesis of bimetallic Ag/Au nanoparticles using orange peel extract and assessing their antibacterial and anticancer potential. J. King Saud. Univ.-Sci..

[55] Khanal L.N., Sharma K.R., Paudyal H., Parajuli K., Dahal B., Ganga G.C., Kalauni S.K. (2022). Green synthesis of silver nanoparticles from root extracts of *Rubus ellipticus* sm. and comparison of antioxidant and antibacterial activity. J. Nanomater..

[56] Annamalai J., Nallamuthu T. (2016). Green synthesis of silver nanoparticles: Characterization determination of antibacterial potency. Appl. Nanosci..

[57] Saxena A., Tripathi R.M., Zafar F., Singh P. (2012). Green synthesis of silver nanoparticles using aqueous solution of *Ficus benghalensis* leaf extract and characterization of their antibacterial activity. Mater. Lett..

[58] Malabadi R.B., Mulgund G.S., Meti N.T., Nataraja K., Kumar S.V. (2015). Antibacterial activity of silver nanoparticles synthesized by using whole plant extracts of *Clitoriaternatea*. Res. Pharm..

[59] Eltarahony M., Zaki S., ElKady M., Abd-El-Haleem D. (2018). Biosynthesis, characterization of some combined nanoparticles, and its biocide potency against a broad spectrum of pathogens. J. Nanomater..

[60] Wang M., Shen J., Thomas J.C., Mu T., Liu W., Wang Y., Liu K. (2021). Particle size measurement using dynamic light scattering at ultra-low concentration accounting for particle number fluctuations. Materials.

[61] Passos M.L., Costa D., Lima J.L., Saraiva M.L.M. (2015). Sequential injection technique as a tool for the automatic synthesis of silver nanoparticles in a greener way. Talanta.

[62] Abdullah A., Annapoorni S. (2005). Fluorescent silver nanoparticles via exploding wire technique. Pramana.

[63] Jian Z., Xiang Z., Yongchang W. (2005). Electrochemical synthesis and fluorescence spectrum properties of silver nanospheres. Microelectron. Eng..

[64] Ahmad N., Seema S., Alam M.K., Singh V.N., Shamsi S.F., Mehta B.R., Anjum F. (2010). Rapid Synthesis of Silver Nanoparticles Using Dried Medicinal Plant of Basil. Colloids Surf. B Biointerfaces.

[65] Kumari R., Negi M., Thakur P., Mahajan H., Raina K., Sharma R., Singh R., Anand V., Ming L.C., Goh K.W. (2024). *Saussurea costus* (Falc.) Lipsch.: A comprehensive review of its pharmacology, phytochemicals, ethnobotanical uses, and therapeutic potential. Naunyn-Schmiedeberg’s Arch. Pharmacol..

[66] Yilma H.G., Fekade B.T., Archana B., Nishant R., Mesfin G., Tadesse A., Nasser S., Kundan K.C., Rakesh K.B. (2023). Anti-inflammatory activity of phytochemicals from medicinal plants and their nanoparticles: A review. Curr. Res. Biotechnol..

[67] Islam M.E., Islam K.M.D., Billah M.M., Biswas R., Sohrab M.H., Rahman S.M. (2020). Antioxidant and anti-inflammatory activity of Heritiera fomes (Buch.-Ham), a mangrove plant of the Sundarbans. Adv. Tradit. Med..

[68] Mutuma G.G., Ngeranwa J., King’ori M.A., Kiruki S. (2020). Phytochemical and Anti-Inflammatory Analysis of Prunus africana Bark Extract. Res. J. Pharmacogn..

[69] Abdelhafez O.H., AliM T.F.S., Fahim J.R., Desoukey S.Y., Ahmed S., Behery F.A., Kamel M.S., Gulder T.A.M., Abdelmohsen U.R. (2020). Anti-Inflammatory Potential of Green Synthesized Silver Nanoparticles of the Soft Coral *Nephthea* Sp. Supported by Metabolomics Analysis and Docking Studies. Int. J. Nanomed..

[70] Gautam H., Asrani R.K. (2018). Phytochemical and Pharmacological Review of an Ethno Medicinal Plant: Saussurea Lappa. Vet. Res. Int..

[71] Gowda R., Dinavahi S.S., Iyer S., Banerjee S., Neves R.I., Pameijer C.R., Robertson G.P. (2018). Nanoliposomal delivery of cytosolic phospholipase A_2_ inhibitor arachidonyl trimethyl ketone for melanoma treatment. Nanomed. Nanotechnol. Biol. Med..

[72] Wang B., Wu L., Chen J., Dong L., Chen C., Wen Z., Hu J., Fleming I., Wang D.W. (2021). Metabolism pathways of arachidonic acids: Mechanisms and potential therapeutic targets. Sig. Transduct. Target. Ther..

[73] Choi Y.K., Cho S.G., Woo S.M., Yun Y.J., Jo J., Kim W., Shin Y.C., Ko S.G. (2013). *Saussurea lappa* Clarke-Derived Costunolide Prevents TNF α -Induced Breast Cancer Cell Migration and Invasion by Inhibiting NF- κ B Activity. Evid.-Based Complement. Altern. Med. eCAM.

[74] Al-Radadi N.S. (2022). *Saussurea costus* for Sustainable and Eco-Friendly Synthesis of Palladium Nanoparticles and Their Biological Activities. Arab. J. Chem..

[75] Groach R., Yadav K., Sharma J., Singh N. (2019). Biosynthesis and Characterization of Silver Nanoparticles Using Root Extract of *Saussurea Lappa* (Decne.) Clarke and Their Antibacterial Activity. J. Environ. Biol..

[76] Alshubaily F.A. (2019). Enhanced Antimycotic Activity of Nanoconjugates from Fungal Chitosan and *Saussurea costus* Extract against Resistant Pathogenic Candida Strains. Int. J. Biol. Macromol..

[77] Bersuder P., Hole M., Smith G. (1998). Antioxidants from a Heated Histidine-Glucose Model System. I: Investigation of the Antioxidant Role of Histidine and Isolation of Antioxidants by High-Performance Liquid Chromatography. J. Am. Oil Chem. Soc..

[78] Berkow E.L., Lockhart S.R., Ostrosky-Zeichner L. (2020). Antifungal Susceptibility Testing: Current Approaches. Clin. Microbiol. Rev..

[79] De A., Albetiza L., François R. (1987). Determination of Phospholipase A2 Activity by a Colorimetric Assay Using a Ph Indicator. Toxicon.

